# An empowerment model of Iranian women for the management of menopause: a grounded theory study

**DOI:** 10.1080/17482631.2019.1665958

**Published:** 2019-10-07

**Authors:** Mansoureh Yazdkhasti, Reza Negarandeh, Zahra Behboodi Moghadam

**Affiliations:** a Department of Midwifery, School of Medicine, Social Determinants of Health Research Center, Alborz University of Medical Sciences, Karaj, Iran; b Nursing and Midwifery Care Research Center, School of Nursing and Midwifery, Tehran University of Medical Sciences, Tehran, Iran; c Department of Reproductive Health, School of Nursing and Midwifery, Tehran University of Medical Sciences, Tehran, Iran

**Keywords:** Empowerment, Menopause, gender Identities, health promotion, grounded theory

## Abstract

**Background and Objectives**: There is still no clear portrayal of women's empowerment in managing menopause. The present study was conducted to design a model for the empowerment of Iranian women in managing menopause.

**Materials and Methods**: This qualitative study was conducted using the grounded theory on  40-60-year-old women who were first selected through purposive sampling and then by theoretical sampling from November 2013 to July 2016. Data were collected using 33 in-depth, semi-structured, individual interviews with 30 participants. Data were analyzed using the Strauss and Corbin(2008) approach and organized in MAXQDA-10.

**Results**: The analysis of the data led to the emergence of "active coping with menopause" as the core variable with four themes. The two themes "threat to feminine identity" and "latent opportunity" explained the context of the study, and "redefining the feminine identity" and "self-retrieval" explained its process.

**Conclusion**: In our social context, the phenomenon of menopause is a coin with two sides and its experience leans more on the threat to feminine identity and less on latent opportunity. The model of postmenopausal women’s empowerment for managing menopause might offer health policy-makers a realistic and divergent understanding of the challenges of empowering women by explaining key concepts.

## Introduction

Menopause is an important event in a woman’s life cycle and its biological process involves the permanent discontinuation of menstruation induced by the cessation of ovarian function and is associated with the lack of menstrual cycles after twelve full months. The natural process of ageing is associated with dramatic biological, psychological and social changes for women (Harris, 2013; Nosek, Kennedy, & Gudmundsdottir, 2012). One of the key aspects of reproductive health is focused on physical, psychological and social health in menopause. The World Health Organization has estimated the number of postmenopausal women to reach 1.2 billion worldwide by 2030. A total of 47 million women go through menopause each year (5). In Iran, 15.78% of the total population will consist of postmenopausal women by 2021 (Simon & Reape, 2009; Yazdkhasti et al., 2012). In the medical model, menopause is described one-dimensionally as a disease and health crisis for women (Kelly, 2011). Meanwhile, the natural phenomenon of menopause is multidimensional, and includes biological, psychological, social, cultural and spiritual dimensions, and is proposed as women’s second maturation (Goldstein, 2000). If menopause symptoms were merely caused by purely-hormonal events, women all over the world would be expected to experience similar symptoms (Nosek et al., 2012). Whereas research findings from many locations indicate that the majority of women pass through the menopausal transition with relatively little discomfort (Lock, 1993). Survey research in the USA and Canada with large samples of women aged 45–55 who are representative of the general population adds substantial support to this finding (Kaufert, Boggs, Ettinger, Woods, & Utian, 1998a; Kaufert, Gilbert, & Hassard, 1988b; McKinlay et al., 1992; Melby, Lock, & Kaufert, 2005). In one study, European women describe menopause as a distressing and health-threatening process (Hall, Callister, Berry, & Matsumura, 2007). In the north Indian culture, women welcome menopause. For these women, menopause is regarded as a natural phenomenon that creates respect, freedom and broader social interactions. These women describe menopause as a sense of appreciation, worth and more in-depth family and social roles (Singh & Arora, 2005).

As opposed to the medical approach, in the feminist view, menopause is a natural, positive and transferring process that gives women a second chance at freedom and is not synonymous with disease (Goldstein, 2000). Feminists believe that menopause is a natural process that can be managed by women’s natural control over their life. They also think that considering menopause a disease turns it into a sensitizing issue and reduces women’s agency (Kelly, 2011).According to the viewpoint of Goldstein, menopause appears to be ambiguous and amorphous; its onset and advancement, the nature and fluctuation of hormonal change, variation in symptom formation, lack of replicability in testing, variation in response to treatment, and ineffability, all de-objectify. Through its resistance to objectification menopause lends itself to metaphorical construction in terms and concepts like deficiency, natural process, and culture-bound syndrome. Then, a change that happens simultaneously with the onset of menopause is the change in the meaning of life (Goldstein, 2000).

In the 1990s, the Ottawa Charter for Health Promotion has made empowerment a key issue in the theory of health-promotion, which focuses on positive health enhancement rather than only ill-health prevention, mainly through the improvement of social conditions (Aujoulat, D’Hoore, & Deccache, 2007; KIckbusch, 2003). By affecting the components of empowerment in postmenopausal women, such as self-efficacy and self-esteem, the change in the meaning of life can lead to reduced self-worth and create a sense of powerlessness and lack of control over the irritating symptoms of menopause (Habibi, 2013). Empowerment is the basis of commitment to health, and people use responsive strategies to solve and manage their problem (Fotoukian, Mohammadi Shahboulaghi, Fallahi Khoshknab, & Mohammadi, 2014). Women’s empowerment is a symbolic marker of the progress of a society. Empowerment is a construct that is interpreted differently by different individuals and is formed in a social context (Abbott & Bernstein, 2015). Successful plans for empowering women to manage menopause requires an understanding of the needs of the target population (Shu, Luh, Li, & Lu, 2007). Women’s traditional gender role is highlighted in Iran’s sociocultural context and the patriarchal and vertical view of women. Iran has a Gender Inequality Index (GII) value of 0.509, ranking it 118 out of 159 countries in the 2015 index (Parvizy, Mirbazegh, & Ghasemzade Kakroudi, 2016).

Given the lack of studies on the empowerment of postmenopausal women and to understand how women are empowered to manage menopause, the present study was conducted to design a model for empowering Iranian women in managing menopause. Health professionals appear to be able to use this model as the basis for more effective interventions and plans for empowering postmenopausal women and promoting their health.

## Materials and methods

### Design

A qualitative method and belief in multiple realities are appropriate for uncovering the nature of people’s experiences (Speziale, Streubert, & Carpenter, 2011). Grounded theory is a qualitative method used to explore social processes and human interactions (Corbin & Strauss, 2008). In this study, the grounded theory approach was used to understand Iranian women’s perspectives on empowerment for menopause management.

### Data collection and participants

The first participants were selected through purposeful sampling (i.e., They had information-rich and in-depth understanding of menopause) and then by theoretical sampling (i.e., data collection arranged to develop conceptual ideas rather than to gather general information). Data were collected from November 2013 to July 2016. The inclusion criteria consisted of being a 40-60-year-old postmenopausal woman (absence of menstrual cycles for at least 12 months), not using hormone therapy in the past six months, not being a case of surgical menopause, no psychological disorders and speaking Persian. First, with the help of the directors of health centres affiliated to Tehran Municipality 128 text messages were sent to the postmenopausal women. 48 women accepted our invitation and assessed for eligibility. Accordingly, 30 postmenopausal women participated in this study. Participants’ written informed consent was obtained. The time and place of the interviews were then decided according to participants’ preferences.

Data were collected mainly through in-depth, semi-structured, face-to-face, individual interview. At first open-ended questions included “how would you describe your experiences of menopause?”What are the positive and negative aspects of the experience?” Then, based on received answers, other interview questions included (1) “How did you come to terms with your menopause and resolve its problems?”, (2) “How were you able to cope with menopausal changes?”, (3) “What factors or conditions contributed to your ability to manage menopause?” and (4) “What factors or conditions impeded your ability to manage menopause?”. Probing questions were also asked, such as “Could you give an example?” At the end of each interview, the participants were asked if they had anything else that they had not discussed. During every interview, we tried to avoid inductive interview questions. Each interview lasted 40–90 minutes, with a mean duration of 65 minutes. To immerse in the data, each interview was carefully listened to and transcribed and typed verbatim. A total of 33 interviews were conducted with 30 participants (Includes 4 health providers and 26 women who were clients of the health system). All the participants were interviewed once, but participants 7, 9 and 23(a health provider) were interviewed twice due to their rich information and the extended duration of the first interview. Each interview was recorded, typed and transcribed prior to the next one, and the analysis of each interview determined the path for the next)i.e., Each interview guided the next one(. Also, we used filed note and Memoing. This process continued until develop categories, dimensions and integrate those categories, and the theory being developed. We stopped sampling when we got theoretical saturation)i.e., The continuation of sampling and data collection until no new conceptual insights are generated).

Consequently, sampling was predominantly theoretical in this study. In this study, maximum variation sampling was considered in terms of age, age at menopause, marital status, education, socioeconomic status, and occupation. Table I presents participants’ details.

**Table I.** The socio-demographic characteristics of the participants.

Characteristic	Number
Age (years)	
Below 50	4
50-55	17
56-60	9
Age at Menopause (years)	
Below 45	6
46-51	17
52-60	7
Marital Status	
Married	14
Widowed	8
Divorced	4
Single	4
Education	
Primary school	6
High school	5
High school diploma	9
Advanced diploma, and bachelor’s degree	10
Socioeconomic Status	
Good	13
Moderate	11
Poor	6
Occupation	
Retired	7
Employed	11
Housewife	12

### Data analysis

Data were analysed using the four stages proposed by Corbin and Strauss (Corbin & Strauss, 2008). In this study, each interview was recorded, typed and transcribed prior to the next one, and the analysis of each interview determined the path for the next. In analysing the data for concept, the researcher carefully reviewed the data line by line to identify the concepts, statements or codes) initial codes and initial categories (. Concepts mean separate subjective labels attributed by the researcher to the phenomenon in question and in-vivo coding has been used to name the concepts. Analysing the data for context was carried out after their analysis for concept in order to establish the link between the categories (initial categories linking). In this stage, the researcher combined data from the first stage, and linked them together in a new form through ongoing comparison. In the stage of bringing process into the analysis, the relationships between the categories were systematically formed (relationships between intra-categories systematically) and integrating the categories. In the stage of integrating the categories, the study’s storyline, core variable, hypothesis and model emerged by way of the relationship formed between the categories and clarification.

Data collection and analyses were carried out by the first author (M.Y) under the supervision of the corresponding author (R.N). The collection process was monitored by the research team in periodical meetings. Analysis and interpretation were carried out by the research team, using Microsoft Word and MAXQDA v. 10.0 to facilitate coding, categorizing, and retrieving data.

### Trustworthiness

The criteria proposed by Lincoln and Guba (2005) were used to ensure the rigour of this study. They included credibility, transferability, dependability and conformability (Denzin & Lincoln, 2005). Credibility was strengthened through prolonged engagement with the samples by two of the researchers (MY and RN), who remained in the process of data collection through to the end. Data rigour was examined with several methods. For credibility and conformability, the allocation of sufficient time to the interviews, prolonged engagement with the data and member check were used. Five member checks were assigned to determine if the codes and categories actually matched participants’ experiences. In the second review, transcription, coding and classification of the codes, concepts and relationships were confirmed by several colleagues as peer check and five participants as member check. Based on the theoretical sampling, participants were selected with maximum diversity in terms of marital status, education, socioeconomic status and occupation. Since discussion and debate are vital to the interpretation of data in qualitative studies, several such meetings were held. All the processes of data analysis were recorded and are available for audit purposes.

### Ethical considerations

The approval of the ethics committee affiliated with the author’s institution (Tehran university of Medical Science) was obtained in November 2013 (decree number = 9121151011–147562). In this research, all the participants were informed of the objectives and methods of the study and gave permission for tape-recording the interviews and publishing the findings. The participants were informed of their rights and the voluntary nature of participation in the study, their anonymity and the confidentiality of their data. They were also ensured that withdrawal from the study was possible without a penalty. Those who wished to participate in the study then signed a written informed consent form.

## Results

In the stage of analysing the data for concepts, 1769 initial codes emerged and following several revisions, combining similar cases and modifications they formed 32subcategories. In the stage of analysing the data for context, the initial categories were linked together to form a logical integration and were divided into 13 subcategories, five main categories and two themes (threat to feminine identity and latent opportunity). In the stage of bringing process into the analysis, intra- categories relationships were systematically formed and 19 subcategories, six main categories and two themes (redefining the feminine identity and self-retrieval) emerged. In the stage of integrating the categories, the study’s storyline, core variable (active coping with menopause), hypothesis and model emerged out of the relationships between the categories and through clarifications (Table II).

**Table II.** Contextual themes, process themes, main categories and core variable.

Contextual Theme	Main Contextual Category	Process Theme	Main Process Category	Core variable
Threat to feminine identity	Understanding the risk of reduced femininity Fear of being different Inadequate support context	Redefining the feminine identity	Change in self-image Acceptance of menopause	Active coping with menopause
Latent opportunity	Changing wisdom Better peace of mind	Self-retrieval	Seeking to advance empowerment Informed self-care Mind control strategies Move towards treatment	

### Threat to feminine identity

Iranian postmenopausal women have shaped their ability to manage menopause in the context of the heavier weight given to the threat to feminine identity and the lighter weight given to latent opportunity.

In 19 interviews of the 33, the threat to feminine identity was an inappropriate context for forming the ability to manage menopause. As one of the contextual themes, the threat to feminine identity emerged from three main categories, including “understanding the risk of losing femininity”, “fear of being different” and “inadequate support”.

The participants were concerned about their family prospects, since menopause means the end of childbearing. Our participants carried the fear of family breakdown and divorce. Participant A remarked:

“A menopausal woman is like a yellow dried leaf. Although menopause happens to all women, a menopausal woman is less fruitful, like a bee with no honey; I mean, she produces no offspring. I feel insecure. My house is like a weak spider web, and I feel like my husband wishes to get rid of me or leave me”.

The end of fertility was an excuse for verbal aggression for the participants. Participant B said:

“When I became menopausal, my husband asked if had become broody (*frowns*), like a chicken that doesn’t lay eggs. He then sneered something under his breath, which I didn’t catch, but annoyed me a lot. I felt humiliated”.

The end of fertility and the vertical and patriarchal views in the society had made them feel old and they feared becoming different. Participant C explained:

“A menopausal woman is misunderstood in our society, as if her use-by date has expired and she’s become useless. This annoys me. Men want happy and young women, not a woman on the slippery slope of ageing. I can no longer satisfy my husband’s sexual desires”.

The end of fertility had caused fear and the loss of hope in marriage, especially in single postmenopausal women. Participant D said:

“I’m still single, and have become menopausal, and have no hope of getting married (*touches her forehead*). Any man getting married would likely want children, and I can’t have kids. I’ve lost my femininity, and I’m different now … . I fear I can’t find a partner for life”.

Although the participants considered menopause a natural phenomenon, given the vasomotor, physical, psychosocial and sexual symptoms, they needed a multifaceted support in the social context; however, the support provided to them was inappropriate and one-dimensional with a merely-medical perspective. Participant F described:

“Ever since I became menopausal, I have visited private clinics and the gynaecological clinic here several times, but they emphasize only a few things, and those are your physical symptoms. They don’t care if you’re distressed about being menopausal. They scare you about the consequences of menopause, but don’t show you a way to have control over your state of mind and mentality. This shows their lack of attention”

### Latent opportunity

The other side of the coin of menopause was experienced as a light-weighing latent opportunity. Latent opportunity was the other contextual theme that emerged out of the two main categories, i.e., change of wisdom and better peace of mind. In 16 interviews of the 33, the participants had experienced a change of wisdom with their menopause.

Participant G explained:

“Ever since my menopause, I reflect more on problems, and use my brain more, so to speak. I see the root of the problem rather than the problem. This has led to fewer mistakes and regrets in my decision-making compared to the past, because my wisdom dominates my feelings and I think more profoundly”.

They also felt relieved since their children had grown up. They associated their children’s independence with an improved peace of mind and freedom in their period of menopause.

Participant H said:

“I’m more comfortable now. I’ve travelled around the world a lot since my menopause, and have enjoyed it very much. Perhaps I was concerned about the children back then and didn't enjoy travelling much”.

Menstrual blood is considered *najes* (ritually unclean) in Islam. The participants therefore felt clean and pure during menopause.

Participant I described:

“Before menopause, every time we went on a pilgrimage, I was worried about getting my period on the trip the whole time, because my periods were irregular. I couldn’t pray (blood is *najes*) or go to the shrine then, but I’m relieved now and feel clean, and no longer have those worries”.

### Redefining feminine identity

Redefining feminine identity emerged as one of the themes in the process of empowerment, with the main categories including change in self-image and acceptance of menopause. Redefining feminine identity consisted of subjective behaviours to avoid the threat to feminine identity to manage menopause.

In 18 interviews of the 33, the participants had experienced menopause as a change of season in a woman’s life and a turning point towards self-awareness and change of self-image.

Participant K said:

“Menopause was a turning point in my life towards myself. I didn’t care much about myself before. For instance, I used to eat whatever I could get my hands on, or if the food burned, I ate the burned parts and gave the top part to my children. I care about myself more since my menopause. I have changed the way I view myself and value myself more now.”

The participants perceived the phenomenon of menopause as a natural event and tried to incorporate it into their feminine identity.

Participant J said:

“Menopause is crystal clear. It’s like the moon; the moon will always rise. It is easier to accept something that happens to everybody. When you accept menopause, then you can handle it”.

### Self-retrieval

Self-retrieval was another theme that emerged in the process of the study and included practical strategies to avoid the threat to feminine identity. Self-retrieval emerged out of four main categories, including seeking to advance empowerment, informed self-care, mind control strategies and moving on the path of treatment.

In 17 interviews of the 33, one of the strategies for seeking to advance empowerment was through enhancing participants’ knowledge. Participant L said:

“I got my bachelor’s degree two years ago. Although my hot flashes were very annoying, my goal was to improve my knowledge. What I mean to say is that knowledge brings power and strengthens you in dealing with menopause”.

Although menopause is a natural event, the need for care was an undeniable part of this process due to its complications, which convinced the participants to manage it by informed self-care. Participant M explained:

“In menopause, I help myself because I feel responsible for my own health. For instance, I walk in the water twice or three times per week to avoid osteoporosis “.

Many participants mentally empowered themselves through prayers, so as to be able to stand the pressures of menopause and gain mind control.

Participant N said:

“I pick up the worry beads and talk to God or say prayers when I’m disturbed on the inside. I become strong and can avoid negative thoughts when I pray”.

Because of the annoying symptoms of menopause, the majority of the participants used a combination of different strategies to control the symptoms of menopause once they had come to terms with it. The participants used a combination of treatments (traditional, herbal and medical). Participant O explained:

“I use phlebotomy to reduce the pain in my bones. Sometimes, I pour salt into a cotton bag, warm it up, and then tie it to my aching legs. I take flaxseed for hot flashes, or drink chicory extract”.

### Active coping with menopause as the core variable for the study hypothesis to emerge

**Figure 1.**
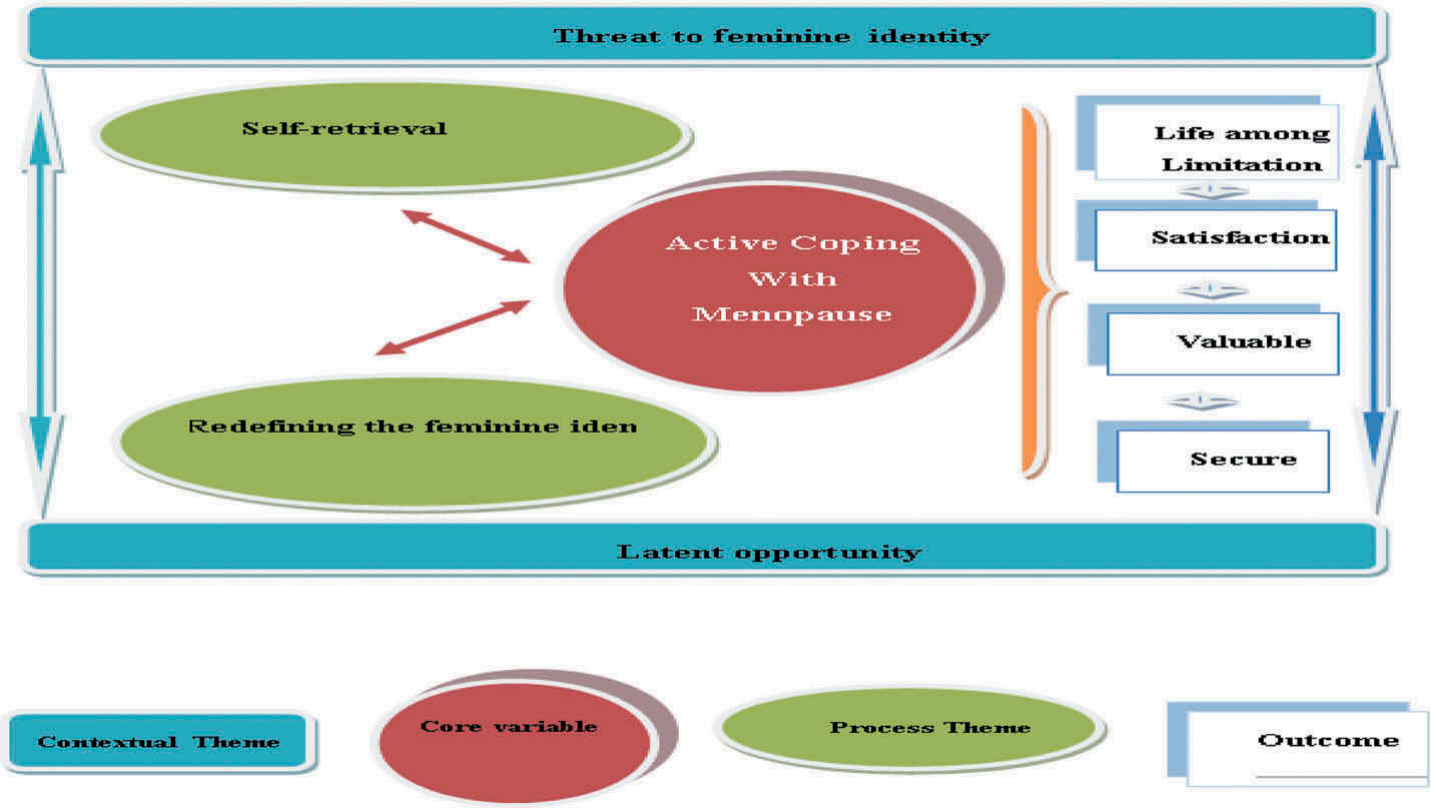
The empowerment model of Iranian women for managing menopause.

In their social context, menopause was experienced by the participants as two sides of the same coin. Their experience of menopause leaned more towards the threat to feminine identity and less to a latent opportunity. To avoid this threat, they had attempted to redefine their feminine identity and gain self-retrieval. Redefining feminine identity initially occurred in their mind by a change in their self-image. At this stage, they achieved self-awareness and valued themselves more. Then, they accepted menopause as a natural event and incorporated it into their feminine identity. In other words, the first links in the process of empowerment to manage menopause that preserved this process was change in self-image and the acceptance of menopause, which took them to the stage of self-retrieval. On this path, they used practical strategies such as seeking to advance empowerment, informed self-care, mind control strategies and move towards treatment, which empowered them to actively face menopause. The outcome of active coping with menopause was living within constraints but feeling secure, valuable and satisfied. The study hypothesis thus emerged (Figure 1): “Through constant effort and active coping, Iranian women were empowered by redefining their feminine identity and achieving self-retrieval within the social context of threat-opportunity and managed their life among limitation within constraints while feeling secure, valuable and satisfied”.

## Discussion

In participants’ experience, the concept of menopause emerged as a double-edged sword. The experience of menopause presented more heavily as a threat to feminine identity and less heavily as a latent opportunity for empowerment. Many studies have also proposed menopause as a double-edged concept. Postmenopausal women in Bangladesh also had a double-edged concept of menopause -indicating both positive and negative changes (Murphy et al., 2013). Swedish women (Lindh-Astrand et al., 2007) experienced menopause both positively (entering a new stage in life) and negatively (feeling old, losing femininity, hormonal changes and the cessation of menstruation). Meanwhile, British-Asian women (Hunter, Bormann, & Lops, 1996) experienced menopause as a triple-edged phenomenon (negative, positive and neutral). Negative experiences (loss of femininity and sinking into loneliness), positive experiences (relief and freedom) and neutral experiences (emerging from concerns about the unknown).

The participants in the present study perceived menopause as a natural event and tried to incorporate it into their feminine identity. A study conducted in East Asia showed that 74% of women consider menopause a natural event and easily accept it (Biri et al., 2005). Women’s acceptance of menopause also depends on men’s attitude. The main concern of Turkish men with regard to menopause was the issue of sex in marital life (Hidiroglu, Tanriover, Ay, & Karavus, 2014). British men also had a negative attitude towards postmenopausal women and did not find their postmenopausal wife capable of suitable and pleasurable sex (Chrisler, Gorman, Marvan, & Johnston-Robledo, 2014). The participants in the present study felt threatened by menopause and saw their status in the family weakened due to Iranian men’s gender-biased attitude, since they could no longer sexually satisfy their husbands as before, and thus carried the fear of family breakdown and divorce. In the experience of women in Northeastern Iran, the husband’s indifference made the women feel worried, insecure and powerless in the family. They constantly feared losing their previous status and being cast aside (Hakimi, Simbar, & Tehrani, 2014).

In Asian countries, including Iran, marriage is affected by traditional gender ideas and cultural barriers, such that childbearing and playing the role of a reproductive woman are essential parts of life that affect the chances of remarriage in single women, infertile women and postmenopausal women and cannot be easily ignored (Abdoli, Ashktorab, Ahmadi, Parvizy, & Dunning, 2011). The results of two qualitative studies quoted by Shepel on middle-aged women (45–60 years old) regarding the middle-age identity showed that single women (widowed or divorced) had experienced a particularly greater departure from their previous identity, which was attributed to their reduced femininity and libido and the end of their childbearing age (Shepel, 2008). In married participants, the end of fertility and the lack of sexual duties had made the husbands less attentive, and some of the husbands had made the women unhappy by using terms such as “brooding” to indicate fruitlessness.

These participants had experienced menopause with a sense of ageing. Some Western women interpret menopause as the powerlessness of women and the loss of attractiveness. They strive to find ways to cope with ageing and insist on looking young (Smirnova, 2012). In most qualitative studies conducted on postmenopausal Iranian women, menopause is described as an unpleasant period filled with feeling older (Hakimi et al., 2014; Rimaz, Zareie, & Shamsalizadeh, 2013), which agrees with the present findings as well.

In the present study, the health providers had a disease-like attitude towards menopause. A study conducted by Seyedfatemi, Salsali, Rezaee, and Rahnavard (2014) showed that Iranian postmenopausal women receive a one-dimensional support from the medical personnel, who regard menopause as a disease and are content with routine training and do not support postmenopausal women in terms of other aspects, such as the psychological aspect (Seyedfatemi et al., 2014). In a study conducted on 24 patients with type-II diabetes, using the strategy of fear to manage diabetes was reported as a barrier to self-care. By frequent visits to physicians, diabetic people saw their own role in managing their diabetes negligible, and this issue was proposed as a barrier to their empowerment for participatory self-care (Abdoli et al., 2011).

In the present study, the other side of the menopause coin was experienced as a latent opportunity for changing wisdom and reaching peace of mind. The women solved their problems and made fewer mistakes in their decisions through their greater intellectual maturity and rational thinking. In some studies, postmenopausal women have been described as experienced, knowledgeable and wise women who are developed from within (Goldstein, 2000; Jones, 1994). Menopause emerged in the experiences of women in Qatar as a life transition and its main categories included “a time for cessation of reproductive obligations”, “a time for managing premenopausal symptoms”, “a time for accepting and celebrating ageing” and “a time for growing into a wise woman” (Murphy et al., 2013). Intellectual maturity has been a positive experience in Turkish women as well that has led to better decision-making and fewer mistakes in life (Cifcili, Akman, Demirkol, Unalan, & Vermeire, 2009). The experience of postmenopausal Iranian women showed that the majority of women consider menopause a period of relief from the engagements of reproduction. They used the terms “wise woman” and “full-grown woman” in their discourse and defined menopause as a second maturity for women (Rimaz et al., 2013). These results agree with those of the present study.

In the present study, the feeling of relief was experienced with a sense of cleanness due to the cessation of bleeding and its resultant “ritually unclean status” that enabled saying prayers and visiting sacred places any time of the month, as, in Islam, a menstruating woman should not pray or visit religious places until her bleeding has stopped. Turkish Muslim women also felt relieved with menopause and this feeling was associated with the end of bleeding and the resultant cleanness that offered the freedom to pray and visit religious places (Cifcili et al., 2009).

In some countries, the fading of the maternal role in postmenopausal women following their children’s independence was not associated with feelings of loneliness, inadequacy and inability (Hall et al., 2007). Unlike the present study, some studies have shown that a lost or diminishing maternal role in postmenopausal women leads to feelings of uselessness, inadequacy, worry and loneliness (Lachmann, 2011).

In the present study, the contextual factors of empowerment to manage menopause were experienced as an opportunity but more specifically as a threat. According to Rappaport’s hypothesis, the process of empowerment is initiated in the absence of despair, threat, lack of control and dependence (Peterson & Zimmerman, 2004). Unlike this hypothesis, subjective and objective/practical empowerment behaviours emerged in the present study as a double-edged phenomenon in a specifically threatening context. Furthermore, some experts believe that the process of empowerment is initiated in a context of insecurity and threat that leads to an awakening and helps start the path of empowerment (Kabeer, 1999). In the present study, the threatening context of feminine identity initiated the process of empowerment. The patriarchal context exposed the participants to a threatening situation, but this issue made them start the process of empowerment with an ongoing and progressive effort. They replaced their self-image with self-worth and respect. A study conducted by Noroozi, Miri, and Mohammadi (2012) on postmenopausal Iranian women based on the PRECEDE model showed that the acceptance of identity as a postmenopausal woman and incorporating menopause into one’s identity are enabling factors that help postmenopausal women have a positive attitude towards menopause and change their behaviour accordingly to manage menopause (Noroozi et al., 2012). In the present study, a changing self-image was the first link in starting the process of empowerment. The acceptance of menopause was the second link in the process of empowerment for managing menopause, and the acknowledgement of menopause as a natural and universal phenomenon helped the participants accept it. In qualitative studies conducted on Indian (Singh & Arora, 2005), Japanese (Lock, 1986) and Qatari Arab (Murphy et al., 2013) women, menopause has been experienced as a positive transitional/developmental process and a natural change of season in life. Western countries’ interpretation of menopause as a disease undermines its natural status in women’s life. With such a perspective, women will be less able to incorporate menopause into their feminine identity (Hall et al., 2007). According to Ryff’s model, mental health is formed as the result of self-acceptance, autonomy, purposeful life, development of personality and domination over the environment, i.e., the individual’s ability to manage and adapt to environmental changes (Ryff & Singer, 2006). In the present study, a changing self-image and the acceptance of menopause (synonymous with self-acceptance in Ryff’s model) were among the first empowerment behaviours in managing menopause and avoiding threat. Personality development (seeking to advance empowerment) and self-retrieval depend on the level of coordination with circumstances and active coping with menopause was consistent with domination over the environment, as proposed in Ryff’s model. Accepting the status quo, accepting the changes caused by menopause and incorporating menopause into one’s identity were the necessary conditions for adopting appropriate strategies for managing menopause in Taiwanese women (Yang, Kenney, Chang, & Chang, 2016). The participants of the present study incorporated menopause into their identity by accepting it and using practical solutions. One of these solutions was to obtain information and knowledge-orientation for managing menopause. Domm, Parker, Reed, German, and Eisenberg (2000) considered access to educational resources as a factor for increasing postmenopausal women’s knowledge and recalled knowledge as a strategy aligned with the process of empowerment (Domm et al., 2000). Studies have used different methods, including individual-based learning (Yazkhasti et al., 2014), group support (Yazdkhasti et al., 2012) and group discussion and empowerment models focused on educating postmenopausal women (Rotem, Kushnir, Levine, & Ehrenfeld, 2005; Yazdkhasti, Simbar, & Abdi, 2015), and have reported a significant increase in postmenopausal women’s awareness in managing menopause and obtaining or promoting empowerment.

Informed self-care was another empowerment behaviour for managing menopause and actively dealing with it in the participants of this study. A self-caring individual is responsible for his own health and reaches self-management through self-care (Soleimani, Negarandeh, Bastani, & Greysen, 2014). In a qualitative study, self-care measures in middle-aged women were addressed using a questionnaire designed based on Orem’s theory of self-care. The results showed that the majority of empowered middle-aged women took the necessary, general, personal and social measures and a quarter of them also took measures related to developmental growth and largely managed menopausal changes (Hartweg, 1993). Another study showed that self-care was more commonly practiced by the more-empowered postmenopausal women (Hermansson & Martensson, 2011).

Prayer is regarded as a type of meditation. Several studies have examined the role of prayers for the acceptance of life crises such as illness (La Roche, 2002). Quoting Neumann, Soleimani et al. (2014) discussed the abundant and important psychological roles of religion and argued that religion helps individuals better understand and cope with life events. Patients with Parkinson’s disease also used prayers to abate the mental pressures of their condition. In this way, they attained peace of mind and fought their illness (Soleimani et al., 2014).

Taking the path of treatment was another practical strategy adopted by the participants to actively cope with menopause. In most western countries, the disease-like view due to female hormone deficiency has led to the use of hormone therapy by postmenopausal women (Potdar & Shinde, 2014). An extensive trial showed that the symptoms of menopause cannot be merely associated with female hormone deficiency; rather, several factors are also involved in the incidence of menopause symptoms. Treating the symptoms of menopause therefore require attention to the psychological, social and cultural problems of postmenopausal women in conjunction with hormone therapy or phytoestrogen therapy (Kelly, 2011; Potdar & Shinde, 2014). The extensive use of herbal remedies in Iran for controlling the symptoms of menopause suggests Iranian women’s greater preference of herbal remedies over hormone therapy (Heydari, Suhrabi, Sayehmiri, & Sayehmiri, 2014). Turkish postmenopausal women had no desire to use hormone therapy to control the symptoms of menopause and controlled menopause with herbal medicines, traditional medicine, mind-control strategies and self-care (Cificili et al., 2009). The participants in this study used a combination of different strategies to control the symptoms of menopause once they had come to terms with it. Along with the obtainment of knowledge, they also used self-care, mind control strategies and herbal or traditional therapies introduced by Avicenna, such as phlebotomy, in order to improve their empowerment. In the present study, following the change in self-image and the acceptance of menopause as mental behaviours targeting empowerment, practical multidimensional strategies used for objective/practical empowerment behaviours also led to the empowerment of women in managing menopause.

## Limitations

The nature of the study limits the ability to generalize the results; however, as with all qualitative studies, the results are not intended to be generalized. Nevertheless, maximum variation sampling was used to select the participants. The researchers acknowledged their own beliefs and views to mitigate the potentially harmful effects of preconceptions that can adversely affect the research process. Prolonged engagement with the participants ensured their trust in sharing their personal experiences.

## Conclusion

The present study showed that Iranian women are empowered to manage menopause in a double-edged context, especially with the threat to feminine identity and through an ongoing effort to actively cope with menopause, and that they experience the process of empowerment on a dynamic and evolutionary path despite the traditional gender view of men, sexual inequality in the society, and the sensitizing nature of menopause. Nonetheless, in actively coping with menopause, Iranian postmenopausal women incorporate menopause into their feminine identity by self-retrieval and by changing their self-image and accepting menopause, and rediscover themselves by practical empowering behaviours. The outcome of such active coping is living within constraints with a sense of security, value and satisfaction.

By recognizing the challenges in the process of empowerment of Iranian women, the model of women’s empowerment for managing menopause appears to have provided health policy-makers with a divergent view for the better understanding of the actual needs of this group, and they can thus adopt more effective plans and strategies for empowering postmenopausal women and promoting their health and quality of life.
